# Comparing Properties of Concrete Containing Electric Arc Furnace Slag and Granulated Blast Furnace Slag

**DOI:** 10.3390/ma12091371

**Published:** 2019-04-27

**Authors:** Jin-Young Lee, Jin-Seok Choi, Tian-Feng Yuan, Young-Soo Yoon, Denis Mitchell

**Affiliations:** 1 Department of Civil Engineering and Applied Mechanics, McGill University, Montreal, QC H3A 0C3, Canada; jinyoung.lee@mcgill.ca; 2 School of Civil, Environmental and Architectural Engineering, Korea University, Seoul 02841, Korea; radiance@korea.ac.kr (J.-S.C.); yuantianfeng@korea.ac.kr (T.-F.Y.)

**Keywords:** electric arc furnace slag, blast furnace slag, initial and final setting, shrinkage, compressive strength

## Abstract

For sustainable development in the construction industry, blast furnace slag has been used as a substitute for cement in concrete. In contrast, steel-making slag, the second largest by-product in the steel industry, is mostly used as a filler material in embankment construction. This is because steel-making slag has relatively low hydraulicity and a problem with volumetric expansion. However, as the quenching process of slag has improved recently and the steel making process is specifically separated, the properties of steel-making slag has also improved. In this context, there is a need to find a method for recycling steel-making slag as a more highly valued material, such as its potential use as an admixture in concrete. Therefore, in order to confirm the possibility of using electric arc furnace (EAF) oxidizing slag as a binder, a comparative assessment of the mechanical properties of concrete containing electric arc furnace oxidizing slag, steel-making slag, and granulated blast furnace (GBF) slag was performed. The initial and final setting, shrinkage, compressive and split-cylinder tensile strength of the slag concretes were measured. It was found that replacing cement with EAF oxidizing slag delayed the hydration reaction at early ages, with no significant problems in setting time, shrinkage or strength development found.

## 1. Introduction 

The steel industry has consumed a great deal of natural materials and energy, with a significant amount of by-products being generated during the steel making process. The fraction of by-products accounts for approximately 50% of steel production. However, most of the by-products are used as filler materials in embankment construction, even though they contain useful components such as iron, carbon, and lime [1,2,3]. 

The main by-products of the steel industry, blast furnace (BF) slag and steel-making (SM) slag, can partially replace cement in concrete because of its potential hydraulicity [1,2,4,5,6,7,8,9]. According to previous studies, about 70% of the total production of BF slag has been used as a replacement for Portland cement due to its numerous advantages, e.g., an increase in long-term strength and durability, decreased heat of hydration and the occurrence of the alkali-aggregate reaction. In contrast, only 1% of SM slag has been used as an admixture because SM slag has relatively low hydraulicity and has a problem with volumetric expansion. However, the chemical composition of SM slag can be controlled by adjusting the steel making process. Recently, the process has been separated into an oxidizing process and a reducing process in order to desulfurize the steel. During oxidation refining, electric arc furnace (EAF) oxidizing slag, which contains lower free-CaO than normal EAF slag, can be obtained [10]. There is a need to find a method that can recycle SM slag as a more highly valued material due to the fact that the annual world production of SM slag is 3.2 billion tons [10]. EAF oxidizing slag still has lower economic benefit than BF slag because steel making plants typically do not have specialized quenching facilities. However, EAF oxidizing slag can be made economically feasible as a useful admixture [11].

A comparative assessment of the mechanical properties of concrete containing electric arc furnace (EAF) oxidizing slag and granulated blast furnace (GBF) slag was performed. The goal was to investigate the effects of the substitution of EAF oxidizing slag and GBF slag for cement, in order to examine the possibility of using EAF oxidizing slag as a binder. In addition, the effects of adding gypsum in the slag concrete was also evaluated to investigate possible improvements in shrinkage behavior and strength. For the experimental program, properties of fresh concrete, e.g., slump, air content, initial and final setting, and shrinkage were measured on six types of concrete specimens. Compressive and split-cylinder tensile strengths were also determined.

## 2. Literature Review

### 2.1. Electric Arc Furnace Slag (EAF Slag)

**Figure 1:**
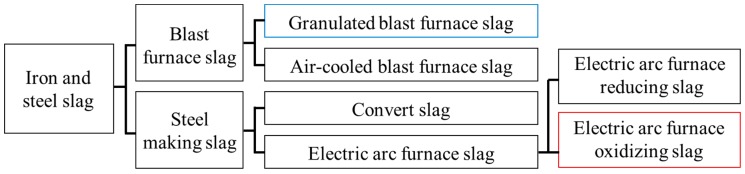
Types of iron and steel slag.

As shown in Figure 1, slags are generally classified into two categories: Blast furnace (BF) slag and steel-making (SM) slag. Electric arc furnace (EAF) slag, one of the SM slags, is produced by refining the process of recycled steel scrap in the electric arc furnace. The main chemical characteristics of EAF slag are its high free calcium oxide (free-CaO) and Fe oxide content. It has been reported that these two compounds cause the volumetric instability of EAF slag. The hydroxylation of free-CaO and subsequent carbonation are accompanied by a significant expansion in volume. In addition, the long-term oxidation of Fe_2_O_3_ to Fe_3_O_4_ is associated with volumetric expansion and the high Fe oxide content reduces the chemical activity during the process of hydration in concrete. For these reasons, EAF slag has not been used as a cementing component. However, as the steel making process is improved, through an oxidizing process and a reducing process, EAF slags can be obtained separately in the manufacturing process. Oxidizing slag is generated during oxidation refining, while reducing slag is generated during a reduction process in order to desulfurize the steel. Quenched EAF oxidizing slag contains low free-CaO because the slag is extracted before the reduction process—which adds CaO into the furnace—and therefore it can have effective hydraulicity when it is finely ground. Recently, several researchers have taken an interest in the possibility of using this slag as a cementitious binder. Shi [12] reviewed several production methods of EAF slag and reported that EAF slag with high basicity and/or subjected to rapid cooling can exhibit a cementing property. Muhmood et al. [2] investigated the cementitious and pozzolanic behavior of EAF slag and confirmed that up to 15% of EAF slag could be substituted for cement in concrete without loss of compressive strength. Zhao et al. [13] performed an experimental study of concrete containing SM slag, focused on the particle size, and reported that the concrete containing properly ground SM slag had good physical and mechanical properties with high durability. Wang et al. [14] also studied the hydration properties of SM slag and showed that the hydration process of steel slag was similar with that of cement. However, its hydration rate was much lower than cement. The hydration rate of steel slag at the early age could be accelerated by raising the fineness of particles, curing temperature or alkalinity of the solution. Zhao et al. [15] revealed the effect of gypsum in the cementitious paste containing SM slag, wherein a similar hydration process with normal cement paste was shown and the addition of gypsum increased the hydration rate at the early age. In summary, the literature confirms the possibility of using EAF slag as a binder and eco-friendly material with high durability [2,10,11,12,14,15]. However, published studies on the material properties of EAF slag concrete are limited, and there is a need for further data, especially on the effect of setting time and shrinkage behavior for practical use.

### 2.2. Granulated Blast Furnace Slag (GBF Slag)

GBF slag is a type of BF slag, which is obtained by water-quenching molten iron slag from the blast furnace. Conversely, if BF slag is air-cooled slowly, it will consist of unreactive crystalline compounds. Approximately 300 kg of BF slag is generated for each ton of steel [10,11]. The many benefits of using GBF slag as a binder in concrete are well known, such as the low heat of hydration, the gain in strength over time and improved durability, as well as environmental advantages [16,17]. Morrow III et al. [18] reported that the environmental benefits focused on CO_2_ emission and energy efficiency with cost analysis. Kim et al. [19] investigated high volume GBF slag concrete and reported that high volume GBF slag replacement—up to 70%—showed an enhancement in the durability and sustainability of concrete. Many research studies have been conducted to investigate the mechanical properties of concrete containing GBF slag, while only a few studies have been carried out on EAF slag concrete [4,5,20,21,22]. Although GBF slag concrete has many benefits, especially in long-term strength and durability [23,24], it also has relatively low initial strength and large autogenous shrinkage that must be taken into account. A number of research studies [25,26] have reported that the addition of alkaline solution or gypsum can increase the initial strength of the concrete and some research studies [7,27] have proposed equations for predicting the shrinkage behavior. 

In this study, specimens of normal concrete and specimens containing GBF slag are used as control specimens for comparison with specimens containing EAF oxidizing slag.

## 3. Experimental Program

### 3.1. Chemical Composition of Materials and Mixture Design

The cementitious materials used in this study were Type 1 Ordinary Portland cement (OPC), EAF oxidizing slag, and GBF slag produced in South Korea. The basic physical properties and chemical composition of the cement and the slags are presented in Table 1. Finely ground OPC and GBF slag, as well as their properties, were provided by the manufacturers, whereas the EAF slag was ground by a ball mill for 3 h until the specific surface area became 5000–5100 cm^2^/g. The fineness of the finely ground EAF slag was measured by using the particle size analyzer CILAS 990. Compositional analysis was conducted by X-ray fluorescence. The T-Fe (FeO and Fe_2_O_3_) and MnO contents in the EAF slag were considerably high, but CaO was relatively low compared to the OPC and GBF slags. 

The details of the mix proportions for the specimens are shown in Table 2. The specimens had a W/B ratio of 0.40 and a sand-to-total aggregate ratio of 0.43. Sea sand and crushed coarse gravel were used as aggregates and the maximum aggregate size was 25 mm.

The design compressive strengths of the OPC and control specimens were 35 MPa at 28 days. For the experimental tests, either 15 or 30% of the Portland cement was replaced by EAF oxidizing slag or GBF slag, as shown in Table 2. The mixes were designated as EAF15, GBF15, and GBF30 based on their replacement ratios. In addition, gypsum was added to EAF15 and GBF30 specimens (i.e., EAF15-G, GBF30-G) in order to investigate the effect of gypsum on the initial strength and the shrinkage behavior.

### 3.2. Test Method

#### 3.2.1. Slump and Setting Properties

It is well known that the substitution of GBF slag for cement improves the workability [28]. However, few studies have reported on the rheological properties of EAF slag concrete. Therefore, a slump test was performed to evaluate the workability of the specimens in accordance with ASTM C143 [29]. In addition, the air content of the mixes was measured in accordance with ASTM C231 [30].

Three cylindrical molds with a diameter of 150 mm and height of 160 mm were prepared for each mix. The standard penetration resistance test was carried out every hour to measure the initial and final setting times of the mixtures according to ASTM C403 [31]. The needle penetrated the specimen to a depth of 25 ± 2 mm in 10 s. The test was conducted at a temperature of 30 ± 1 °C and a relative humidity of 60 ± 5%.

#### 3.2.2. Autogenous and Dry Shrinkage

Six prismatic specimens (100 mm × 100 mm × 100 mm) for each mixture were cast, and each set of three specimens was tested to examine the autogenous and drying shrinkage responses. A dumbbell-shaped strain gage with nearly zero stiffness and a thermocouple was cast horizontally in the middle of each mold. A Teflon sheet was placed inside the molds to prevent any friction between the molds and the concrete. For the specimens that measured autogenous shrinkage, the upper surface of the specimen was sealed with a polyester film right after concrete casting to prevent moisture loss. After 24 h, the specimens were demolded and immediately sealed with aluminum adhesive tapes. For the specimens that measured drying shrinkage, the specimens were tested without sealing them. The first data acquisition began approximately 1 h after casting, and data collection continued for 35 days. All specimens were stored and tested in a constant temperature and humidity room of 23 ± 1 °C and 45 ± 5%, respectively.

#### 3.2.3. Compressive and Split-Cylinder Tensile Strength 

The compressive strength at 3, 7, and 28 days was measured for each mix by three concrete cylinders (diameter 100 mm and height 200 mm) according to ASTM C39 [32]. In addition, the standard test for splitting tensile strength of cylindrical concrete specimens was also carried out at 3, 7, and 28 days after casting in accordance with ASTM C496 [33].

## 4. Test Results and Discussion

### 4.1. Slump and Setting Properties

As can be seen in Table 3, the EAF15 mixture shows relatively low slump and air content, even though the replacement ratio of cement with EAF oxidizing slag is not large. When the slumps of GBF15 and GBF30 were compared with that of OPC, the same or slightly higher values of slump were measured. These differences in slump between EAF and GBF mixtures can be caused by the difference in particle size of the binders. The addition of fine EAF slag decreases the air content of the concrete mixture and could result in a decrease of fluidity. The addition of gypsum decreases the slump of fresh concrete, because the accelerated hydration reaction by gypsum can affect the fluidity of the mixtures. 

In order to prevent water evaporation from the surface of the specimens, the specimens were covered with a water-impermeable cover during the penetration test in accordance with ASTM C403. To determine the initial and final setting times, Equation (1) was used for regression analysis.

(1) Log(PR)=a+bLog(t)

where PR is the penetration resistance in MPa, *t* is the elapsed time in hours, and *a* and *b* are the regression constant and coefficient, respectively.

**Figure 2:**
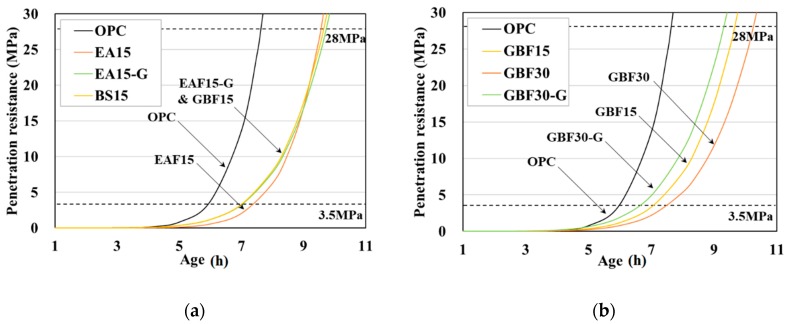
Setting times of different types of concrete. (**a**) Results of EAF series and (**b**) results of GBF series.

The regression constant, *a*, coefficient, *b*, coefficient of determination, *R*^2^, and initial and final setting times are summarized in Table 4. The initial and final setting times were defined using a penetration resistance criteria of 3.5 MPa and 28 MPa, respectively, in accordance with the standard. Curves of penetration resistance and time are shown in Figure 2. It can be seen that cement substitution with slags delayed the initial and final setting times. The initial setting time of OPC, the control specimen, occurred 5.95 h after casting. A comparison of the setting times of EAF15 and GBF15, which have the same replacement ratio, indicated that the EAF oxidizing slag concrete had a shorter setting time. In the case of the GBF series, as the amount of substitution increased, the setting times were further delayed. The addition of gypsum in both the EAF and GBF slag concrete specimens resulted in earlier setting times.

### 4.2. Autogenous and Drying Shrinkage 

Autogenous shrinkage is defined as the macroscopic bulk deformation of cementitious materials by hydration of cement, not subjected to external forces. It is strongly affected by the water-binder ratio and admixtures [27]. Drying shrinkage is the contracting of a hardened concrete mixture due to the loss of capillary water. This type of shrinkage is dependent on the amount of water and admixture, as well as the drying conditions while curing.

**Figure 3:**
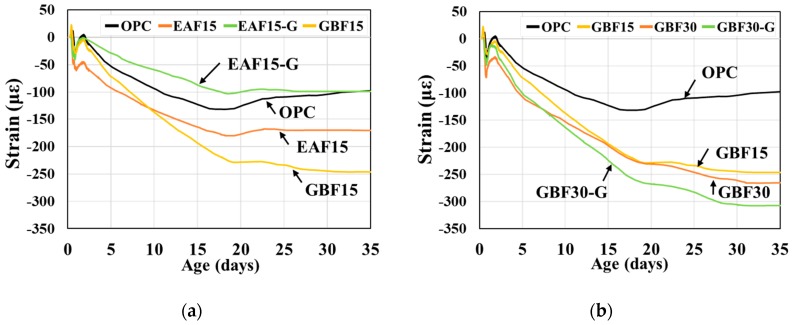
Results of autogenous shrinkage. (**a**) Results of EAF series and (**b**) results of GBF series.

**Figure 4:**
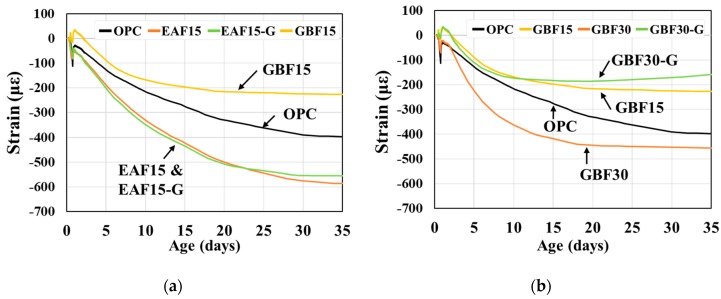
Results of drying shrinkage. (**a**) Results of EAF series and (**b**) results of GBF series.

The autogenous and drying shrinkage of the OPC and slag concretes is illustrated in Figure 3 and Figure 4. As previous research reported [27,34], both slag concretes showed greater autogenous shrinkage than OPC. The slope of the shrinkage curves converged to zero after 20 days in the case of the OPC and EAF series. For the GBF series, the shrinkage curves converged to the final values after 30 days and greater autogenous shrinkage is observed for the GBF series compared to the EAF 15 series. The addition of gypsum decreased the shrinkage of the EAF concrete but increased the shrinkage of the GBF concrete. These results may be associated with the amount of latent hydration materials in the concretes. The hydration reaction, which increased autogenous shrinkage, occurred in the GBF concrete after 7 days up to 28 days, whereas most of the hydration reaction occurred in the EAF concrete at an early age.

### 4.3. Compressive and Split-Cylinder Tensile Strength 

**Figure 5:**
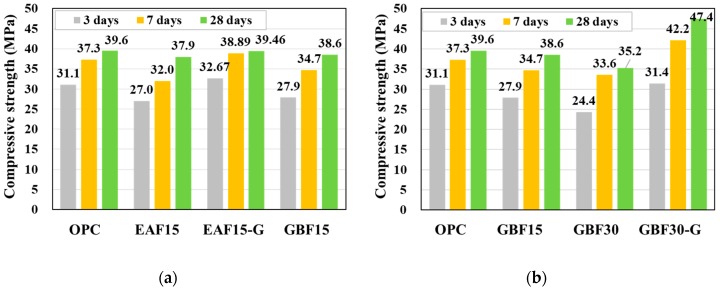
Compressive strength. (**a**) Results of EAF series and (**b**) results of GBF series.

The compressive strength at 3, 7, and 28 days after casting are given in Table 5 and in Figure 5. It can be seen that both the compressive strength of EAF and GBF slag concretes developed more gradually when compared with the strength gain of OPC. However, the strength ratios at 28 days were 96% and 98% for the EAF15 and GBF15, respectively. Therefore, it can be concluded that most of the hydration reaction is completed before 28 days and a significant decrease in the compressive strength has not occurred when the small amount of cement—i.e., 15% in this study—is replaced with either EAF or GBF slags. Moreover, it was observed that the addition of gypsum accelerated the compressive strength development at early ages.

When the cement was replaced with 30% GBF slag, the strength development was delayed at the early ages and the strength ratio was 88% at 28 days. However, the latent hydraulic capacity of the GBF slag can be utilized by the addition of gypsum (GBF30-G), resulting in increased compressive strength at 28 days.

**Figure 6:**
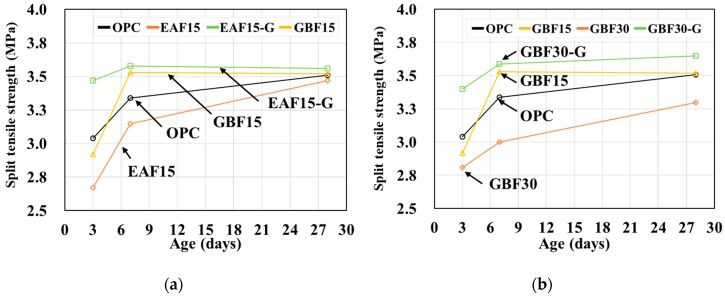
Split-cylinder tensile strength. (**a**) Results of EAF series and (**b**) results of GBF series.

The split-cylinder tensile strengths at 3, 7, and 28 days after casting are shown in Table 6 and Figure 6. The trend of the tensile strength development was similar to that of the compressive strength development. The rates of tensile strength development of slag concretes were slower than that of OPC. However, the strengths of slag concretes at 28 days were similar to the strength of OPC, except for GBF30. 

## 5. Conclusions

Initial and final setting, autogenous shrinkage, drying shrinkage, compressive and split-cylinder tensile strength of EAF and GBF slag concretes were evaluated to examine the possibility of using EAF oxidizing slag as a binder. Based on the above results, the following conclusions can be made:

(1) The properties of fresh concrete were evaluated by measuring the slump and air content. The mixtures substituted with EAF oxidizing slag for cement showed a decrease in the slump with lower air content when compared with the results of OPC. In contrast, GBF slag concrete showed similar results with that of OPC. These differences of slumps between EAF and GBF mixtures were caused by the difference in the particle size of binders. The addition of fine EAF slag decreases the air content of the concrete mixture, potentially resulting in a decrease of fluidity.
(2) Using EAF oxidizing slag and GBF slag as a substitute for cement delayed the initial and final setting times. The addition of gypsum slightly shortened the setting times of the fresh concrete, but it did not make a significant difference at very early ages. It was found that the delayed setting times of EAF mixtures were not very different from the setting times of the GBF mixtures. This is a positive result for EAF slag because GBF slag is already widely used as a substitute for cement.
(3) Both slag concretes showed greater autogenous shrinkage than OPC. The slope of the shrinkage curves converged to zero after 20 days in the case of OPC and the EAF series. For the GBF series, the shrinkage curves converged to the final values after 30 days.
(4) The compressive and split-cylinder strengths at 3, 7, and 28 days were measured. The strength development of the EAF and BGF slag concretes was slower than that for OPC at early ages, but there was not a significant decrease in the strength at 28 days when 15% of cement was replaced with EAF oxidizing slag. In addition, it was observed that the addition of gypsum accelerated strength development. Therefore, it may be suggested that the use of EAF oxidizing slag as a cementitious binder did not cause problems in either shrinkage behavior or strength when a small amount of oxidizing slag was substituted for cement.

## Figures and Tables

**Table 1:** Physical properties and chemical composition of the cement and slags.

Type	Specific Surface (cm^2^/g)	Density (g/cm^3^)	Chemical Composition (%)							
			SiO_2_	CaO	Al_2_O_3_	T-Fe *	MgO	SO_3_	MnO	TiO_2_
OPC	3400	3.15	22.0	64.2	5.5	3.0	1.5	2.0	-	-
EAF slag	5050	3.60	16.1	20.6	12.0	37.3	4.4	-	5.6	0.7
GBF slag	4250	2.90	34.2	45.1	14.3	0.5	3.9	0.2	0.2	0.7

* FeO, Fe_2_O_3_.

**Table 2:** Mix designs of specimens.

Variables	W/B	S/a	Unit Weight (kg/m^3^)						
			W	C	FA	CA	EAFS	GBFS	Gypsum
OPC	0.40	0.43	226	562	598	786	-	-	-
EAF15	0.40	0.43	226	478	598	786	84	-	-
EAF15-G	0.40	0.43	226	478	598	786	84	-	12
GBF15	0.40	0.43	226	478	598	786	-	84	-
GBF30	0.40	0.43	226	394	598	786	-	168	-
GBF30-G	0.40	0.43	226	394	598	786	-	168	12

W/B: Water to binder ratio; S/a: Sand-total aggregate ratio by weight = FA/(FA + CA); W: Water; C: Cement; FA: Fine aggregate; CA: Coarse aggregate; EAFS: Electric arc furnace oxidizing slag; GBFS: Granulated blast furnace slag.

**Table 3:** Results of slump and air content.

Type	OPC	EAF15	EAF15-G	GBF15	GBF30	GBF30-G
Slump (mm)	180	120	106	180	189	170
Air content (%)	4.0	2.7	3.0	3.8	3.6	3.9

**Table 4:** Results of initial and final setting.

Type	*a*	*b*	*R* ^2^	Initial Set (h)	Final Set (h)
OPC	−5.934	8.366	0.984	5.95	7.63
EAF15	−6.778	8.384	0.995	7.47	9.57
EAF15-G	−5.011	6.533	0.990	7.08	9.74
GBF15	−5.091	6.639	0.984	7.06	9.66
GBF30	−5.308	6.684	0.994	7.51	10.25
GBF30-G	−4.482	6.120	0.997	6.63	9.31

*a*: regression constant, *b*: regression coefficient; *R*^2^: coefficient of determination.

**Table 5:** Results of compressive strength.

Type	3 Days		7 Days		28 Days	
	Mean (MPa)	C.V. (%)	Mean (MPa)	C.V. (%)	Mean (MPa)	C.V. (%)
OPC	31.06	2.32	37.25	4.54	39.57	13.43
EAF15	26.98	4.29	31.96	2.50	37.93	0.63
EAF15-G	32.67	14.83	38.89	0.36	39.46	2.41
GBF15	27.86	6.71	34.66	1.21	38.58	6.67
GBF30	24.69	1.71	33.60	0.36	35.24	12.11
GBF30-G	31.44	1.33	42.17	4.10	47.40	5.17

Mean: mean value of compressive strength; C.V.: coefficient of variation.

**Table 6:** Results of split-cylinder tensile strength.

Type	3 days		7 days		28 days	
	Mean (MPa)	C.V. (%)	Mean (MPa)	C.V. (%)	Mean (MPa)	C.V. (%)
OPC	3.04	2.46	3.34	15.07	3.51	15.20
EAF15	2.67	4.73	3.15	10.95	3.47	2.16
EAF15-G	3.47	4.76	3.58	11.75	3.56	7.148
GBF15	2.74	17.14	3.57	8.82	3.52	6.75
GBF30	2.81	16.54	3.00	8.97	3.30	16.55
GBF30-G	3.71	16.11	3.59	16.42	3.65	25.06

Mean: mean value of compressive strength; C.V.: coefficient of variation.
